# Liver tumor boundaries identified intraoperatively using real-time indocyanine green fluorescence imaging

**DOI:** 10.1007/s00432-016-2267-4

**Published:** 2016-09-14

**Authors:** Ya-Min Zhang, Rui Shi, Jian-Cun Hou, Zi-Rong Liu, Zi-Lin Cui, Yang Li, Di Wu, Yuan Shi, Zhong-Yang Shen

**Affiliations:** Department of Hepatobiliary Surgery, First Central Hospital, No. 24 Fukang Road, Nankai District, Tianjin, 300192 China

**Keywords:** Indocyanine green, Fluorescence imaging, Liver neoplasms, Hepatectomy

## Abstract

**Purpose:**

Clear delineation between tumors and normal tissues is ideal for real-time surgical navigation imaging. We investigated applying indocyanine green (ICG) fluorescence imaging navigation using an intraoperative administration method in liver resection.

**Methods:**

Fifty patients who underwent liver resection were divided into two groups based on clinical situation and operative purpose. In group I, sizes of superficial liver tumors were determined; tiny tumors were identified. In group II, the liver resection margin was determined; real-time navigation was performed. ICG was injected intravenously at the beginning of the operation; the liver surface was observed with a photodynamic eye (PDE).

**Results:**

Liver resection margins were determined using PDE. Fluorescence contrast between normal liver and tumor tissues was obvious in 32 of 35 patients. A boundary for half the liver or specific liver segments was determined in nine patients by examining the portal vein anatomy after ICG injection. Eight small tumors not observed preoperatively were detected; the smallest was 2 mm.

**Conclusions:**

ICG fluorescence imaging navigation is a promising, simple, and safe tool for routine real-time intraoperative imaging during hepatic resection and clinical exploration in hepatocellular carcinoma, enabling high sensibility for identifying liver resection margins and detecting tiny superficial tumors.

## Introduction

The development of a real-time intraoperative detection system that is sensitive and specific for tumors will help ensure complete tumor resection, as a clear boundary between tumor and normal tissues is ideal for real-time surgical imaging, particularly for tiny (<5 mm) liver metastases and superficial liver tumors that cannot be observed before surgery (van der Vorst et al. 2012). Although sensitive optical sensors and probes have been developed, including nanoparticles, fluorescent proteins, or near-infrared (NIR) fluorescent molecules, time is necessitated for these entities to be used routinely in clinical practice (de Chermont et al. 2007; Adusumilli et al. 2006; Veiseh et al. 2007).

As a surgical navigation technology, an indocyanine green (ICG) fluorescence imaging (FI) system has had a positive effect on the surgical treatment of gastric cancer, breast cancer, and skin cancer, as well as other tumors (Miyashiro et al. 2008; Kitai et al. 2005; Tsujino et al. 2009; Kurihara et al. 2015; Morita et al. 2013). Further, ICG is used in liver function tests and accumulates in hepatocellular carcinoma (HCC) tissues postintravenous injection (Shibasaki et al. 2015). In 2009, Ishizawa et al. reported the first application an ICG–FI navigation system in the surgical treatment of liver tumors (Lim et al. 2014). Moreover, ICG is a relatively safe reagent that is clinically approved by the United States Food and Drug Administration (FDA) for examination of hepatic function, cardiac output, and retinal angiography and has been used for these purposes for 20 years (Lim et al. 2014; Kitai et al. 1999). Given that ICG is already used in clinical imaging examinations, the use of ICG in intraoperative imaging may help improve the accuracy of tumor and liver tissue demarcation and identification.

In the present study, we introduced an efficient ICG–FI system that allows for accurate navigation during liver resection using an intraoperative ICG delivery method in China.

## Materials and methods

This study was approved by the Institutional Review Board of the Tianjin First Central Hospital.

### Research objective

Fifty patients underwent liver resection between Sep 2014 and May 2015 at Tianjin First Central Hospital. The presence of focal liver lesions was confirmed with abdominal ultrasound, enhanced computed tomography (CT), or magnetic resonance imaging (MRI), and distant involvement and metastases were ruled out. Liver function was assessed to determine the Child-Pugh score, and an ICG skin test was performed before surgery.

According to the actual tumor status and operative goal, patients were divided into two groups: group I, determination of the range in size of superficial tumors and identification of a small tumor; and group II, determination of a liver resection margin and real-time navigation during a liver resection.

### Patient inclusion criteria

To be enrolled in this study, patients had to meet the following criteria: confirmed lesion occupying the liver using abdominal ultrasound, abdominal CT scan, and MRI plus four-phase CT imaging before surgery; exclusion of distant involvement and metastasis; Child A liver function during a preoperative assessment; did not have embolization treatment before surgery; and negative ICG skin test before surgery.

### Experimental reagents and equipment

In this study, we used an ICG injection (Dandong Medical and Pharmaceutical Co, Ltd). According to the manufacturer’s instructions for the ICG solution, it was used at a concentration of 2.5 mg/ml, and light exposure was avoided. Further, a photodynamic eye (PDE, Hamamatsu Photonics Trading Co, Ltd) was used.

### Research methods

In this study, we had a group comprising patients who underwent the following: determination of the range in size of liver superficial tumors and identification of small tumors. For the ICG procedures, we used the following technique. First, an intravenous injection of ICG at a 0.25-mg/kg dose was administered during surgery through the following routes: (1) portal vein puncture; (2) right vein of the stomach; and (3) central venous catheter. Second, an infrared fluorescence observation camera was turned on, using the imaging mode on the camera. Third, we adjusted the brightness, contrast, and sharpness of the photon eye. Fourth, a surgeon used a satirized package of lenses and cables for the photon eye. Fifth, the surgeon irradiated the liver with the photon eye at a distance of 5–10 cm above the liver surface. Lastly, PDE equipment was used to directly examine the liver, so the liver cancer could be detected; subsequently, the cutting edge of the liver could be confirmed. After surgery, the specimens were sent to pathology, and the intraoperative observations were compared with the pathological findings of the liver.

In this study, we had a second group that underwent the following: determination of hepatic resection margin and real-time navigation during a hepatectomy. To do so, the following procedures were performed. (1) The precut half of the liver was marked using the following methods: Intraoperatively, the hepatic portal vein was dissected to free it, along with its left and right branches. The portal vein branches in the resected liver were ligated. ICG was infused at a dose of 0.25 mg/kg through a central venous catheter. PDE was used to clearly observe the line at which the liver would be cut in half. Then, the liver resection line was marked in the PDE approach guide.

We used the following methods to mark a standard cut segment of the liver. According to the preoperative imaging findings, the left and/or right pedicle of the liver was dissected and freed intraoperatively. Then, the liver parenchyma was removed using Cut-ultrasound aspiration (CUSA), and the Glisson’s sheath of the segments or subsegments was fully visualized. The portal vein was freed, and the distal portal vein on the precut side was clamped. After injection of 1–2 mg of ICG through the puncture site and release of the vessel clip, PDE allowed for the clear observation of the status of the liver segment and was used to determine the hepatectomy line.

## Results

### Characteristics of the study subjects

A total of 50 patients were included in this study, with 30 men and 20 women, and their mean age was 54.18 years. There were 16 (32 %) patients aged 40–50 years, 21 (42 %) patients aged 50–60 years, and 13 (26 %) patients aged 60–75 years. The enrolled patients had the following characteristics: 38 had hepatitis B, and 5 had hepatitis C.

There were 35 (70 %) patients in group I (small tumors group; determination of the range in size of superficial tumors and identification) and 15 (30 %) in group II (liver resection group; determination of the liver resection margin and real-time navigation) (Table 1).

**Table 1:** Characteristics of study subjects

	*N*	%
Age		
40–50	16	32.0
50–60	21	42.0
60–75	13	26.0
Sex		
Male	30	60.0
Female	20	40.0
Tumor position		
1 hepatic segment	7	14.0
2 hepatic segments	16	32.0
>2 hepatic segments	27	54.0
Postoperative pathology		
Hepatocellular carcinoma	38	76.0
Cavernous hemangioma	4	8.0
Cholangiocarcinoma	3	6.0
Colon cancer with liver metastasis	2	4.0
Malignant fibrous histiocytoma	1	2.0
Micronodular cirrhosis	2	4.0
Group		
Group I	35	70.0
Group II	15	30.0

Variables in Table 1 are shown as counts and percentages

Group I: determination of ranges in size of superficial tumors and identification of small tumors

Group II: determination of liver resection margin and real-time navigation in liver resection

The patients had the following tumor sizes: ≤3 cm in 6 patients, >2 cm and ≤5 cm in 16 patients, and >5 cm in 28 patients. As for tumor position, the tumor was confined to 1 hepatic segment in 7 (14 %) patients, involved 2 liver segments in 16 (32 %) patients, and involved more than 2 liver segments in 27 (54 %) patients. Regarding the overall postoperative pathology, 38 (76 %) patients had hepatocellular carcinoma, 4 (8 %) patients had a cavernous hemangioma, 3 (6 %) patients had a cholangiocarcinoma, 2 (4 %) patients had colon cancer with liver metastases, 1 (2 %) patient had a malignant fibrous histiocytoma, and 2 (4 %) patients had micronodular cirrhosis (Table 1).

### Effects of different ICG administration routes on liver FI with PDE

**Fig. 1:**
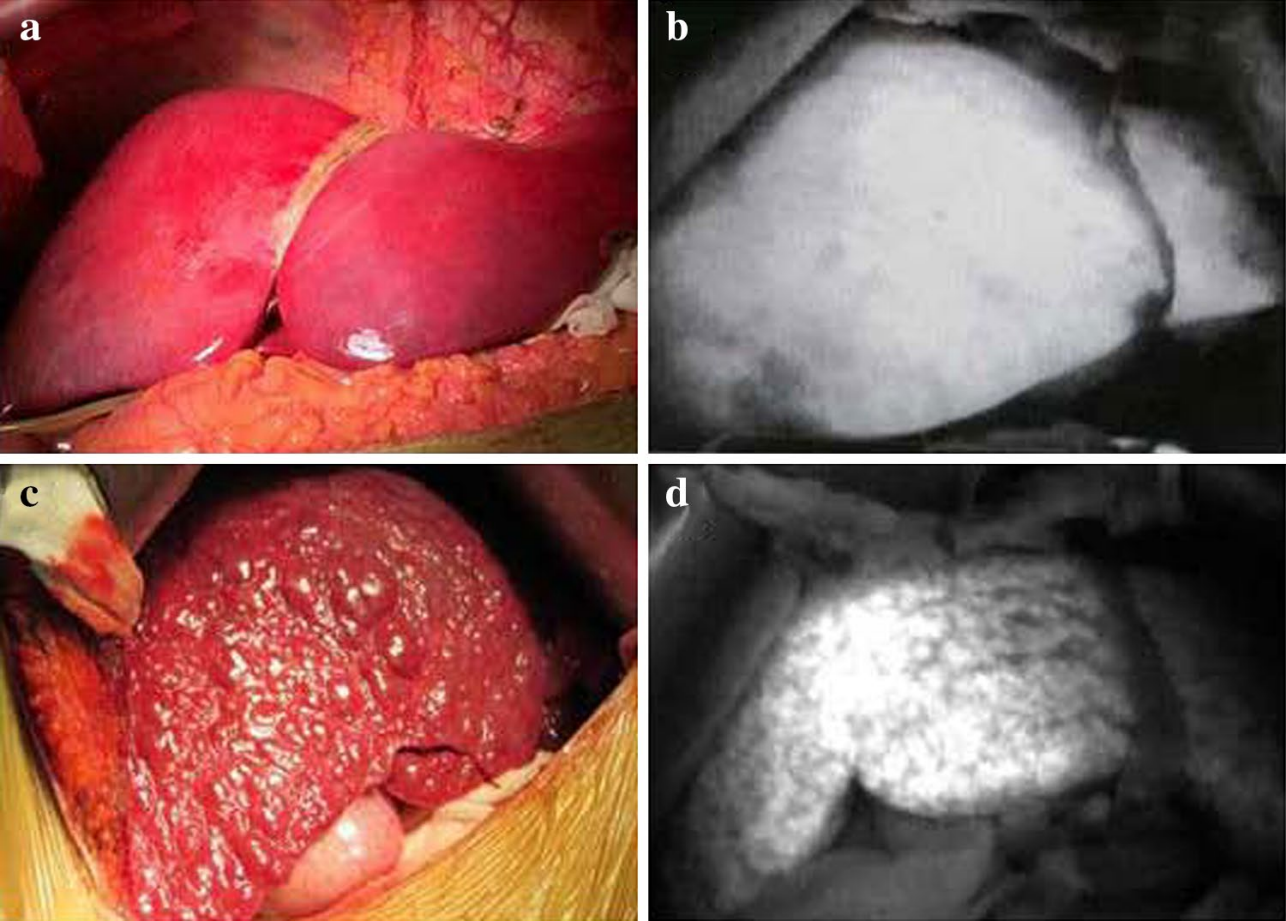
Differences in imaging findings between a normal liver and cirrhotic liver with a photodynamic eye after the intraoperative administration of *indocyanine green*. Normal liver (**a**); normal liver with uniform fluorescence as seen with a photodynamic eye (**b**); cirrhotic liver (**c**); cirrhotic liver with uneven fluorescence as seen with a photodynamic eye (**d**)

With an ICG injection through the portal vein or right vein of the stomach, fluorescence of the normal liver developed rapidly within 1–2 min, while fluorescence of the cirrhotic tissue developed slowly and was not uniform (Fig. 1). With an ICG injection through the central venous catheter, fluorescence of the normal liver developed in 5–10 min and was uniform, and the fluorescence pattern observed with this route of administration was not different from that observed with administration through the portal vein or the right vein of the stomach.

### Effects of intraoperative administration method on liver tumor fluorescence imaging under PDE

**Fig. 2:**
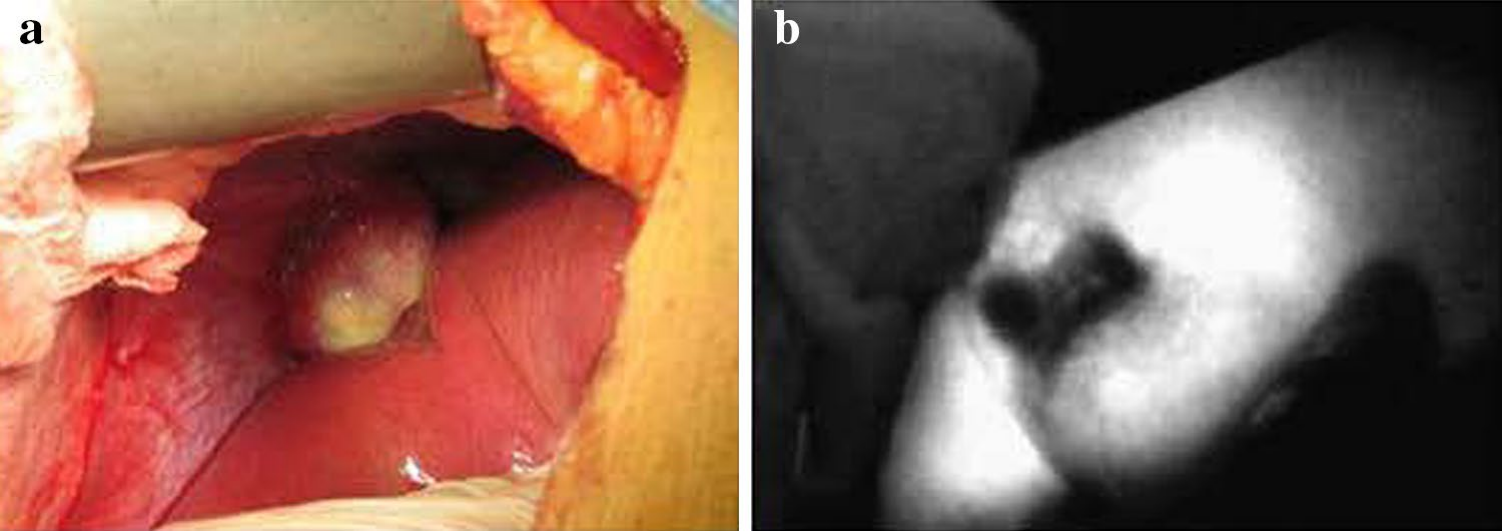
Changes to the liver after being suppressed by a tumor as seen with a photodynamic eye. A patient with a diaphragmatic tumor exerting downward pressure on the liver with no invasion (**a**), the liver under pressure as visualized with a photodynamic eye (**b**)

**Fig. 3:**
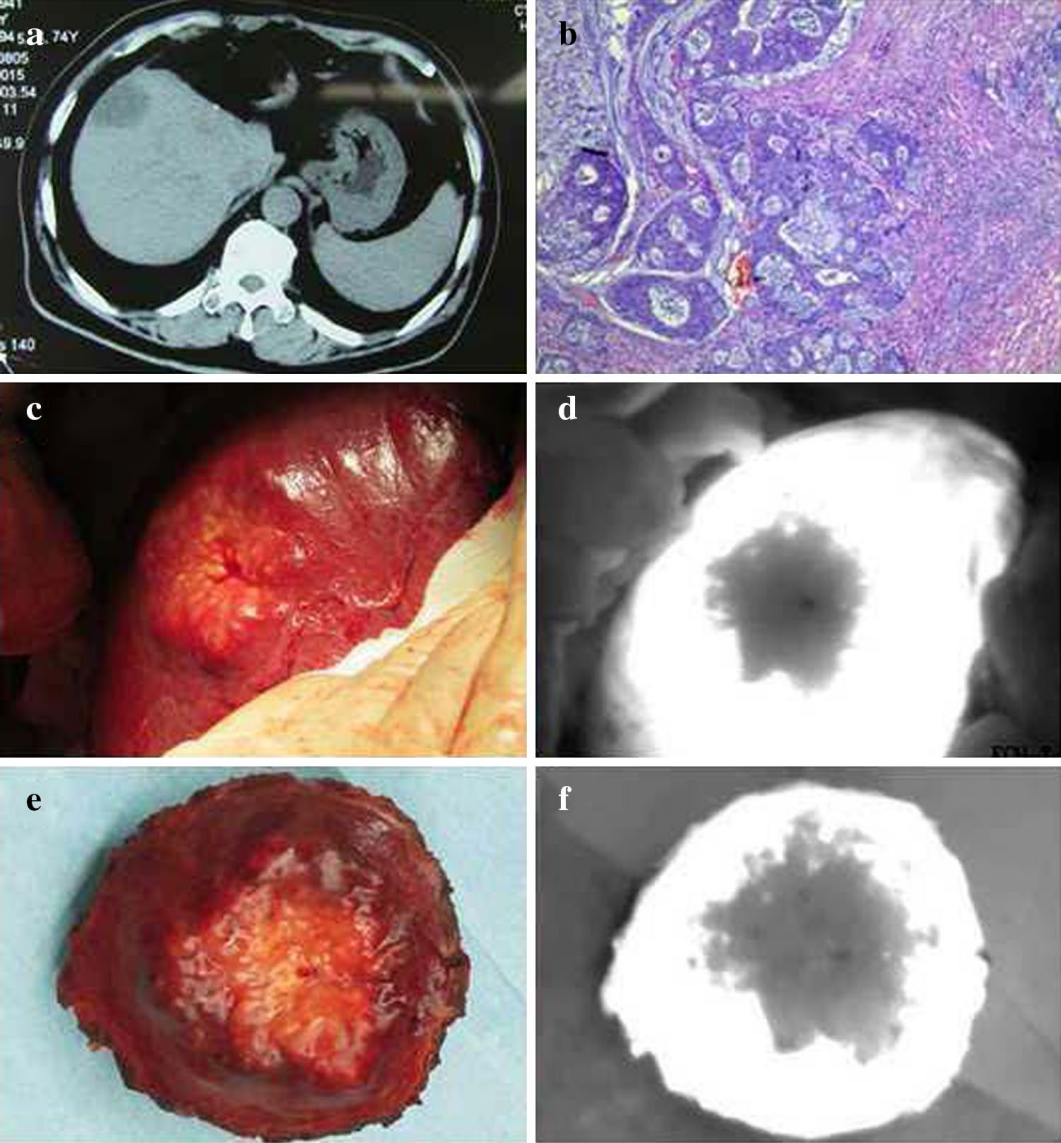
Intraoperative *indocyanine green* administration for visualization of liver metastases with a photodynamic eye. Preoperative computed tomography showing colon cancer with liver metastasis in the right lobe (**a**), pathologic diagnosis of colon cancer metastasis (**b**), visible liver tumor during surgery (**c**), no tumor development visualized with a photodynamic eye, and the boundaries between the tumor and normal liver were clear (**d**), surgical resection specimens (**e**), inspection of specimen with a photodynamic eye (**f**)

**Fig. 4:**
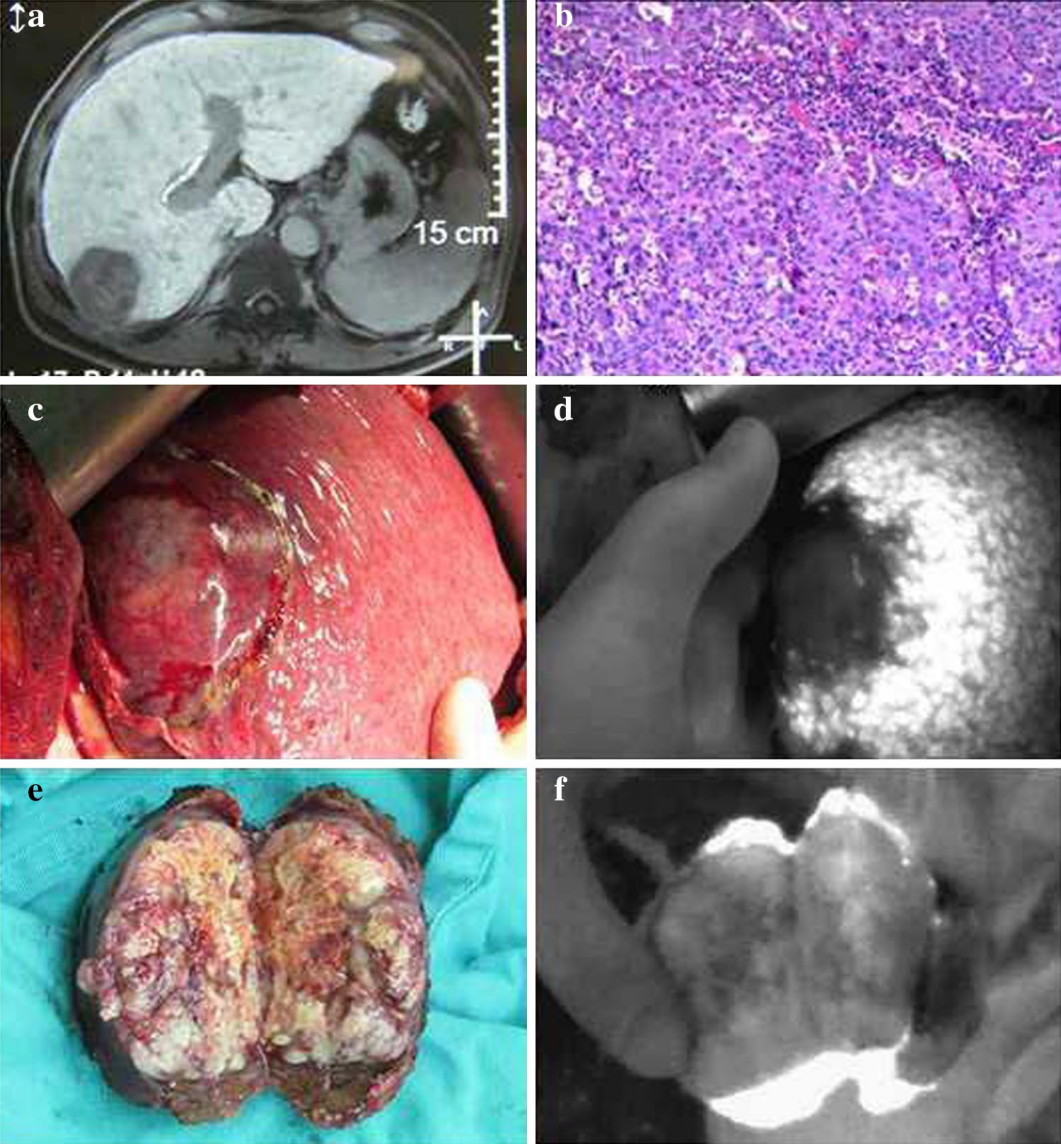
Intraoperative *indocyanine green* administration for visualization of hepatocellular carcinoma with a photodynamic eye. Preoperative magnetic resonance imaging showing liver cancer in the right lobe (**a**), pathologic diagnosis of hepatocellular carcinoma (**b**), tumor viewed intraoperatively (**c**), no tumor development along with clear tumor boundaries, as visualized with a photodynamic eye (**d**), after surgery, cutting the specimen revealed that the excised tumor had a complete capsule (**e**), inspection of a section with a photodynamic eye revealed no tumor development (**f**), there was visible fluorescence surrounding the normal liver tissue, demonstrating that the tumor tissue cannot quickly absorb *indocyanine green*, showing intraoperative fluorescence

After administration of the drug, tumors appeared as a shadow with a PDE, as compared with normal liver tissues. This difference was obvious; the fluorescence pattern was consistent; and no differences existed in the fluorescence patterns between benign and malignant tumors (Figs. 2, 3, 4). The postoperative pathological findings were as follows: HCC (23 patients), micronodular cirrhosis (2 patients), cavernous hemangioma (4 patients), malignant fibrous histiocytoma (1 patient), cholangiocarcinoma (3 patients), and colon cancer with liver metastasis (2 patients). The margins of all liver resection specimens were negative.

### Identification of small liver tumors with fluorescence imaging with a PDE

**Fig. 5:**
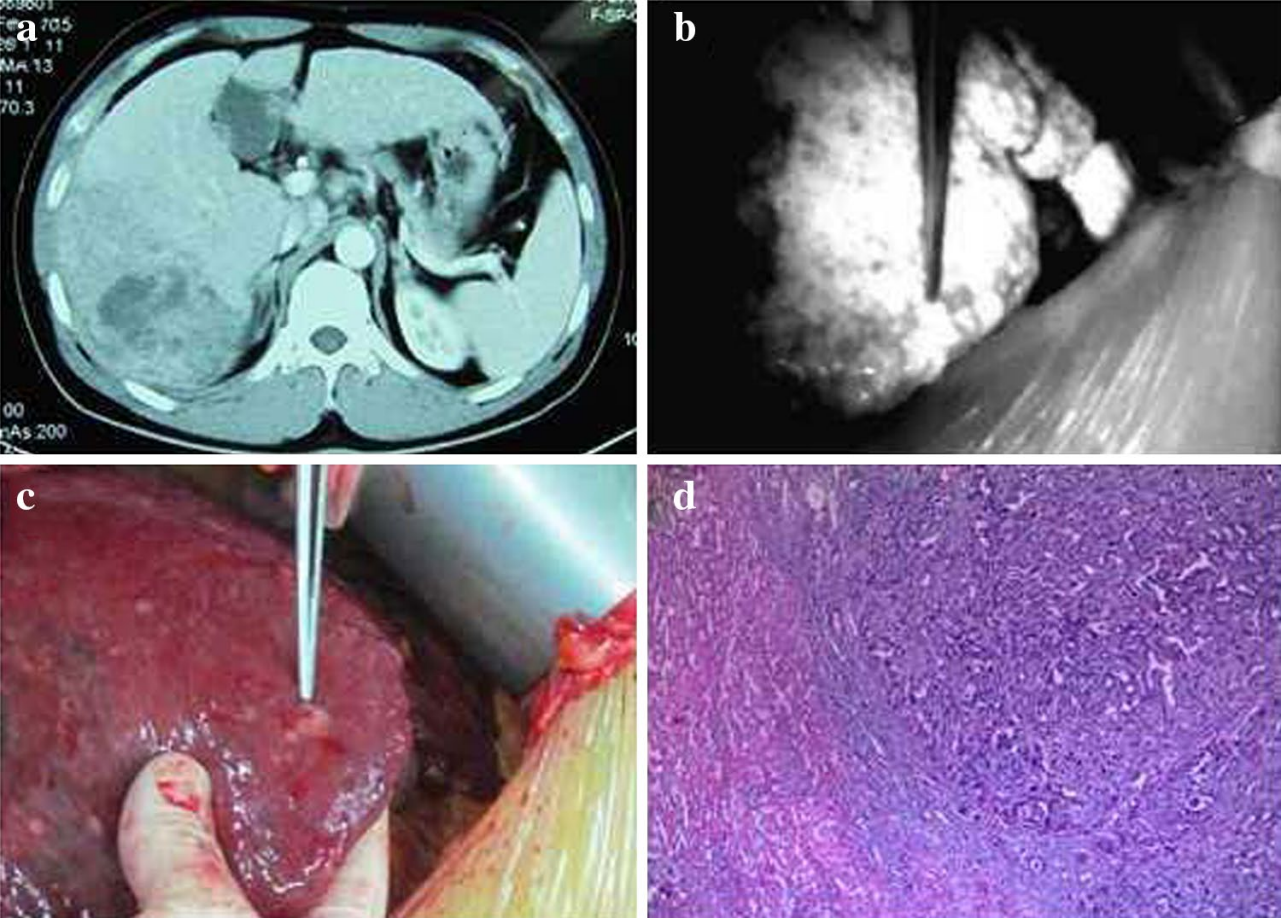
Application of a photodynamic eye for identifying tiny tumors with intraoperative *indocyanine green* administration. Preoperative computed tomography showing a visible VII segment of a liver tumor, and the other segments had no obvious small tumor foci (**a**), intraoperative photodynamic eye examination of the liver; in addition to known tumors, a 4-mm diameter shadow nodule was visible (**b**), tumor nodules were found on the liver surface and sent for pathological analysis (**c**), pathological results confirming hepatocellular carcinoma (**d**)

With the use of an intraoperative PDE, we found 12 small tumors in 8 patients in whom a preoperative imaging examination did not reveal the existence of these tumors. The postoperative pathological findings were as follows: hepatocellular carcinoma (five patients), recurrent nodular cirrhosis (two patients), liver macrovesicular steatosis (one patient), hemangioma (two patients), and hepatic focal hyperplasia (two patients). The smallest lesion was approximately 2 mm in diameter (Fig. 5).

### Determination of the liver resection margin or hemihepatectomy line using FI with a PDE

**Fig. 6:**
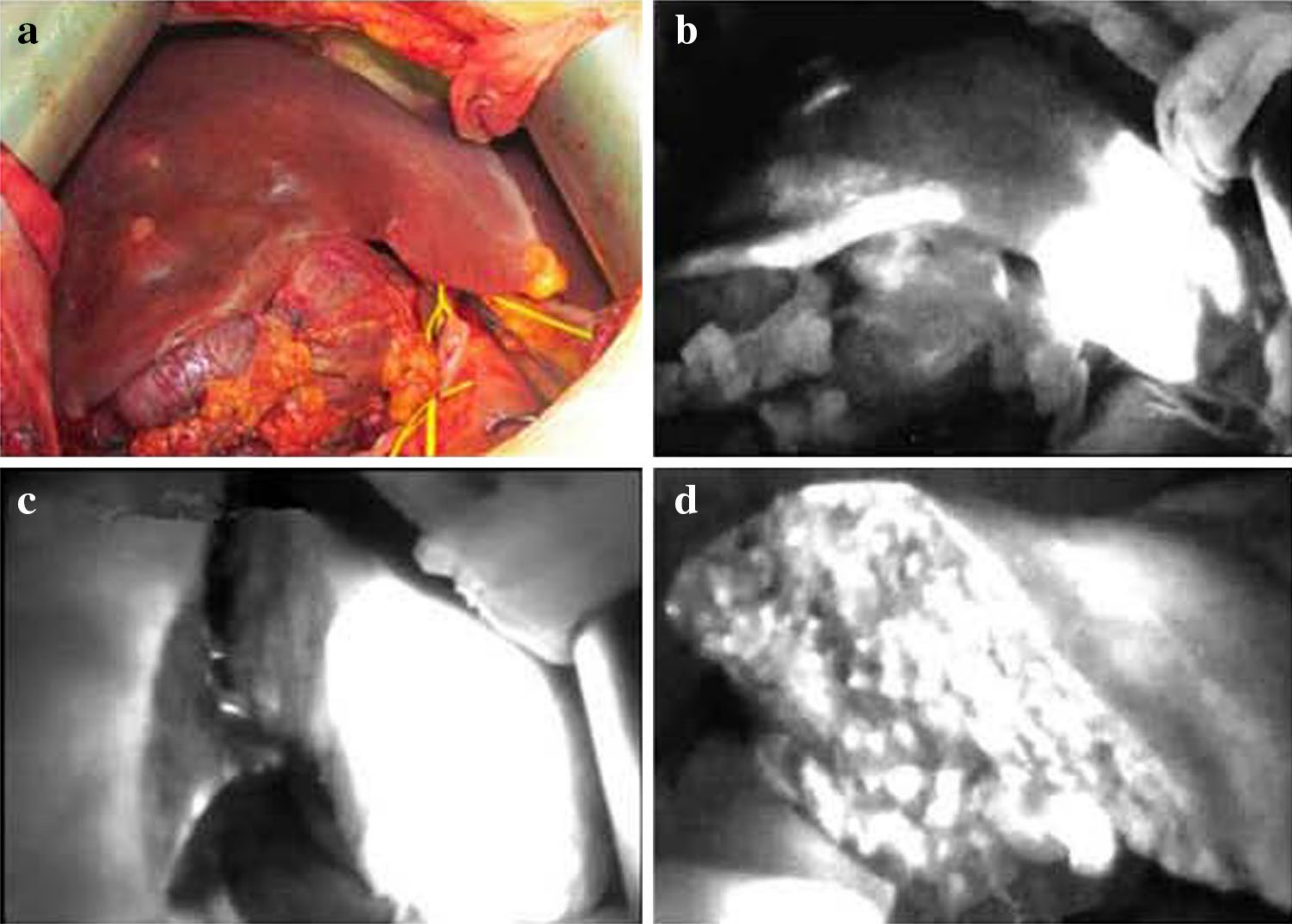
Semi-hepatectomy with the assistance of a photodynamic eye. After ligation of the right hepatic artery and right back branch of the portal vein, the right hepatic ischemia line was not obvious (**a**); after administering *indocyanine green*, fluorescence rapidly developed in the left hepatic lobe, and visualization with the photodynamic eye clearly and accurately revealed the hepatectomy line (**b**). Then, the liver was split along that line; the liver during the splitting process (**c**). A photodynamic eye can be used at any time to observe the cutting route so it can be adjusted if necessary; resection of one-half of the liver is complete (**d**); examination of the liver sections with the photodynamic eye, the remaining liver showed full fluorescence, indicating that the resection met the requirements of complete semi-hepatectomy

Five to 10 min after an ICG injection through a central venous catheter, hemihepatic fluorescence of the precut side was observed, and the hemihepatectomy line was seen clearly and accurately with a PDE. The liver was cut along this line, and the line was monitored with a PDE during surgery to allow the direction of cutting to be altered if necessary (Fig. 6).

## Discussion

In the present study, using an intraoperative administration of ICG with an FI navigation system and a PDE, we found 12 small tumors in 8 patients in whom a preoperative imaging examination did not indicate that these tumors existed. Of these eight patients, five patients had hepatocellular carcinoma; we also found the following conditions: recurrent nodular cirrhosis (two patients), liver macrovesicular steatosis (two patient), hemangioma (two patients), and hepatic focal hyperplasia (two patients). With our intraoperative ICG–FI navigation system, the smallest detected tumors were approximately 2 mm in diameter.

ICG is an FDA-approved reagent that is safe for examination of hepatic function, cardiac output, and retinal angiography (Lim et al. 2014; Kitai et al. 1999). ICG is a near-infrared fluorescent dye that is stimulated by light wavelengths of 750–810 nm that produce near-infrared light with a wavelength of 850 nm (Porcu et al. 2016). Near-infrared light can be observed using an ICG–FI system with a PDE and is displayed with the help of a developing device (van der Vorst et al. 2012; Porcu et al. 2016; Abo et al. 2015).

ICG fluorescence can penetrate living tissue deeply and thus can be advantageous for visualizing these tissues (Gotoh et al. 2009). In a preliminary study, Kitai et al. reported that fluorescence was observed from an ICG solution embedded 1 cm deep in material with optical properties compatible with human tissues; after an intravenous injection of ICG, it was rapidly ingested by the liver, as indicated by the stimulation of emission light (Miyashiro et al. 2008). To that end, after several hours, the liver can completely excrete ICG, and the fluorescence gradually dissipates (de Graaf et al. 2011). However, in cases of liver cirrhosis, liver regeneration nodules, liver cancer, and other types of liver dysfunction, the secretion and excretion function of liver cells is impaired, and ICG remains in lesions; thus, the dissipation of fluorescence is delayed (Gotoh et al. 2009; de Graaf et al. 2011; van der Vorst et al. 2013).

ICG–FI is a safe and minimally invasive technique (Gotoh et al. 2009). Several studies have reported that an intravenous ICG administration of 0.25–0.5 mg/kg from 12–24 h to 14 days before surgery helps to identify tumors with clear boundaries with intraoperative FI (Tsujino et al. 2009; Morita et al. 2013; Lim et al. 2014; Kawaguchi et al. 2013). In a report by Kawaguchi et al., an 81-year-old man with recurrent HCC secondary to hepatitis C was administered an ICG injection 4 days prior to surgery that revealed clear fluorescence of the tumor on the liver surface during surgery (Kawaguchi et al. 2013). In the present study, fluorescence contrast between normal liver and tumor tissues was obvious in 32 of 35 patients with intraoperative ICG administration. A boundary for half the liver or specific liver segments was determined in nine patients by examining the portal vein anatomy after ICG injection.

At present, the mechanisms that mediate the accumulation of ICG in HCC nodules relative to normal liver tissue remain unknown. Normal liver tissue can rapidly uptake ICG, and ICG is usually eliminated in bile; however, severely cirrhotic liver tissue may not be able to eliminate ICG (Sear 1990; Verbeek et al. 2012). In contrast, it has been shown that ICG passively accumulates in HCC if ICG is administered 1–8 days prior to surgery; therefore, borders of superficial liver tumors can be visualized clearly and accurately with a PDE (Verbeek et al. 2012). Using this principle, a boundary of a tumor can be determined that can aid in the determination of the liver resection line.

Three intraoperative routes of administration of ICG were clinically validated in this study. Administering ICG into the portal vein or right vein of the stomach, fluorescence of normal liver tissue developed within 1–2 min, while fluorescence of the cirrhotic tissue developed slowly and non-uniformly. However, administering ICG through a central venous catheter, fluorescence of normal liver tissue developed uniformly in 5–10 min, but the fluorescence pattern observed with this route of administration did not differ from that observed with administration through the portal vein or right vein of the stomach. Moreover, central venous ICG administration resulted in slightly slower and weaker fluorescence, but with an increased dose, the results significantly improved. We observed that central venous administration avoided the anatomy of the portal hepatic and right gastroepiploic veins, shortening the operative time and reducing injuries.

An ICG fluorescent system has the ability to detect small lesions that preoperative imaging examinations cannot help identify (van der Vorst et al. 2012). Preoperative imaging examinations such as CT and ultrasound have limited detection rates for small tumors in the liver (van der Vorst et al. 2012). Even with intraoperative ultrasounds, tiny lesions on the liver surface can be easily missed, leading to the occurrence of liver metastasis in the short term. With preoperative ICG administration, it can be difficult to detect a demarcation line in the liver parenchyma, as cirrhotic liver nodules have the same appearance as tumor tissues (Kurihara et al. 2015). At this time, one negative is that malignant tumors cannot be distinguished from benign tumors, thus leading to a high false positive rate (Morita et al. 2013). In this study combining ICG imaging with the use of a PDE, eight patients had small tumors that were not identified during a preoperative imaging examination, including five patients who had malignant lesions.

Because of more accurate preoperative imaging assessments and intraoperative real-time three-dimensional navigation instructions, liver resection has become more accurate (Morise et al. 2014). It can also be used repeatedly, avoiding the disadvantages of repetitive dyeing of the liver with methylene blue (Ishizawa et al. 2012; Ishizawa and Kokudo 2013; Lim and Vibert 2013).

This study had some limitations. First, this study had a small sample size. Additional investigation with a larger sample size is necessitated to demonstrate these results. Second, this study did not compare the results demonstrated in the two groups with those of a comparator group. Third, this study did not compare the current results with those of prior studies. Therefore, future study is needed to compare preoperative ICG injection timing and various routes of ICG administration and to further apply this tool in clinical practice.

## Conclusion

The current findings indicate that an ICG–FI navigation technology is a new real-time intraoperative imaging method. This technique is promising for hepatic resection and clinical exploration in HCC. Our study demonstrated that intraoperative ICG–FI navigation enables the identification of small and grossly unidentifiable liver cancer tumors in real time, enhancing the accuracy of liver resection and operative cancer staging.
